# Polarization and Phase Textures in Lattice Plasmon Condensates

**DOI:** 10.1021/acs.nanolett.1c01395

**Published:** 2021-06-02

**Authors:** Jani M. Taskinen, Pavel Kliuiev, Antti J. Moilanen, Päivi Törmä

**Affiliations:** Department of Applied Physics, Aalto University School of Science, P. O. Box 15100, Aalto, FI-00076, Finland

**Keywords:** plasmonics, nanophotonics, Bose−Einstein condensation, surface lattice resonance, surface plasmon polariton

## Abstract

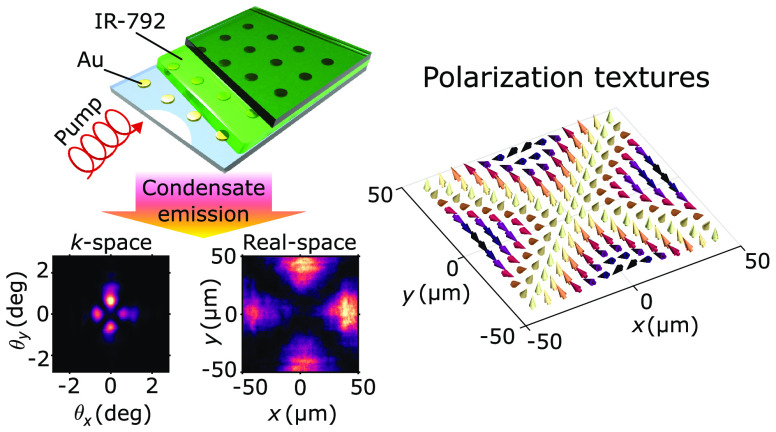

Polarization textures of light may reflect fundamental phenomena, such as topological defects, and can be utilized in engineering light beams. They have been observed, for instance, in photonic crystal lasers and semiconductor polariton condensates. Here we demonstrate domain wall polarization textures in a plasmonic lattice Bose–Einstein condensate. A key ingredient of the textures is found to be a condensate phase that varies spatially in a nontrivial manner. The phase of the Bose–Einstein condensate is reconstructed from the real- and Fourier-space images using a phase retrieval algorithm. We introduce a simple theoretical model that captures the results and can be used for design of the polarization patterns and demonstrate that the textures can be optically switched. The results open new prospects for fundamental studies of non-equilibrium condensation and sources of polarization-structured beams.

Phase transitions and spontaneous symmetry breaking are often associated with topological defects, for example vortices with windings of the phase of a superfluid or superconductor. A vector field with a (pseudo)spin or polarization degree of freedom allows an even richer set of topological defects such as skyrmions, merons, half-vortices, nodal lines, and magnetic monopoles. These have been observed in solid state,^1^ liquid crystal,^2^ ultracold gas,^3^ and liquid helium systems,^4^ as well as in polarization textures of light emerging from polariton condensates.^5−9^ Polarization textures are also widely applied in beam engineering, lasing, and holography.^10−14^

Three main approaches are typically used when creating polarization textures of light: (1) spontaneous appearance in a phase transition or a quench, (2) imposing the texture via an excitation or pump beam, and (3) advanced structural engineering of the medium supporting the optical modes. Polariton condensates in semiconductor systems are amenable for the first two; however, polarization textures there have been observed only at cryogenic temperatures, and complex structural engineering (e.g., see refs (15−17)) is technically demanding. The latter is well developed in traditional photonic crystals and metamaterials, which, when combined with a gain medium, show lasing. However, strong-coupling condensation phenomena with related interactions have not been reported in those systems. Here, we introduce a novel way of creating polarization textures. It is based on a remarkable finding that we report here: the Bose–Einstein condensate (BEC) hosted by the lattice has a nontrivial spatially varying phase profile. For the first time for any kind of condensate, we reconstruct the BEC phase by a phase retrieval algorithm, avoiding interference measurements. We introduce a model that shows how the non-uniform phase, the lattice nanoparticle dipoles, and the polarization of the light pumping the condensate can be used for the design and switching of desired patterns. We experimentally demonstrate this new concept in a simple geometry and show that it leads to domain wall formation. Our system provides an extremely easy and versatile engineering of the geometry and unit cell of the lattice,^18−21^ along with room temperature strong-coupling condensation leading to effective interactions,^22,23^ and ultrafast sub-picosecond operation.^23,24^ The demonstrated new approach to polarization texture creation combines these assets in an unprecedented way and is expected to be fruitful both in fundamental studies of non-equilibrium condensation phenomena^25^ and in beam polarization engineering,^11−13^ particularly when compact and ultrafast components are desired.

An illustration of the system and the experiments is shown in Figure 1a. A square array of golden nanoparticles fabricated on a glass substrate is immersed in a fluid and sealed with a cover glass. The fluid is either an index-matching oil for studies of the bare array or a fluorescent dye solution such as a gain medium for the lasing and condensation measurements. The bare arrays support collective plasmonic modes called surface lattice resonances (SLRs), which are hybrid modes consisting of the localized surface plasmon resonances of individual nanoparticles and the diffracted orders of the periodic lattice.^19,20^ Excitations in the SLR modes are bosonic quasiparticles that have a mainly photonic nature but also consist of collective electron oscillations in the nanoparticles; for their dispersion, see Figure 1b. The Γ-point of the dispersion provides a band edge that may host lasing or condensation.

**Figure 1:**
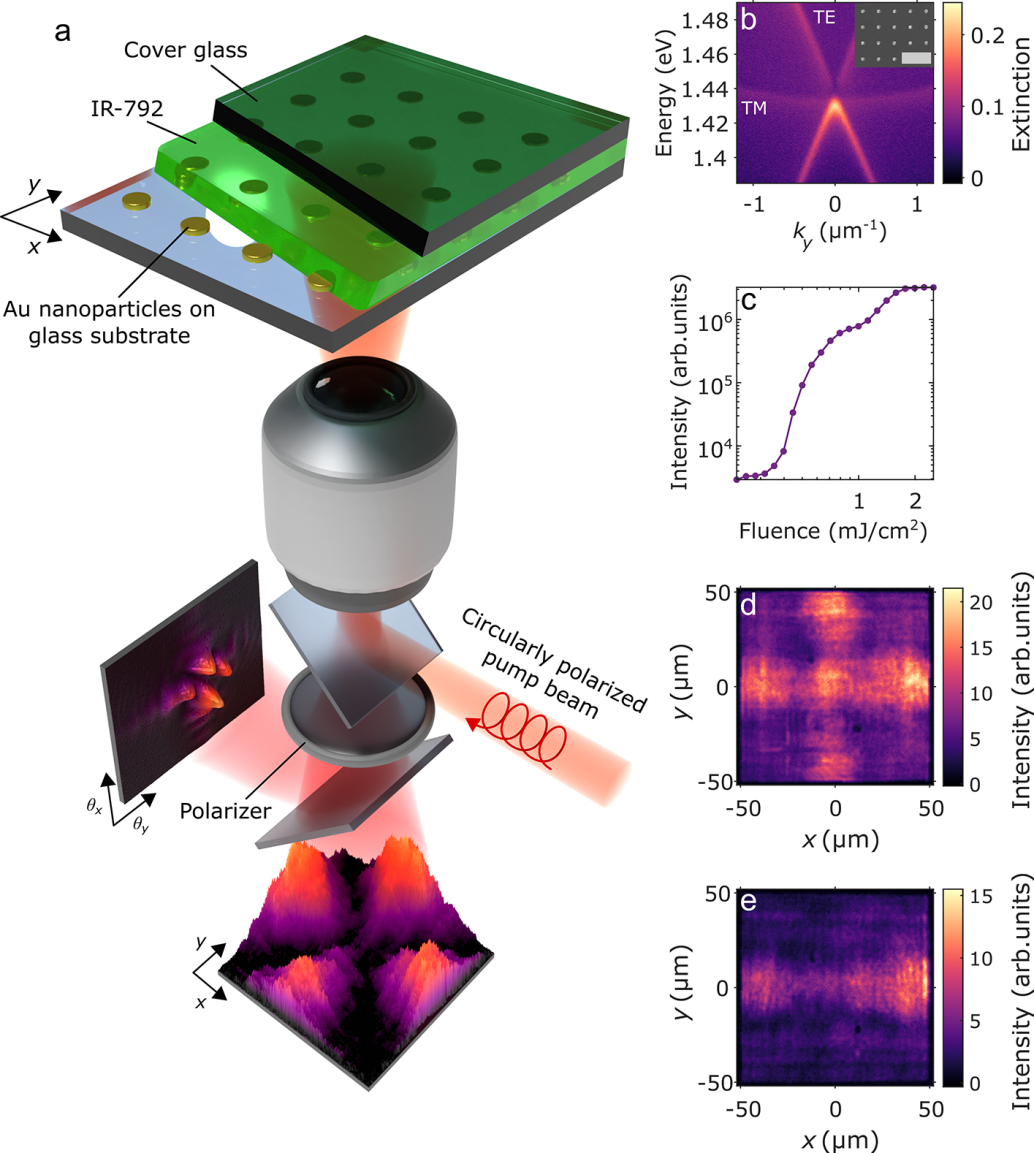
Spatially non-uniform condensation of lattice plasmon excitations. (a) Illustration of the sample structure and the experimental configuration. Both the pumping of the molecules (IR-792) on the sample and collection of the sample photoluminescence are done via the same objective; see Figure S1 and related discussion in the Supporting Information for further details. (b) Extinction of a nanoparticle array without molecules immersed in index-matching oil measured as (1 – *T*), where *T* is the transmission. Here *k*_*y*_ corresponds to the wave vector component parallel to the sample surface and is related to the emission angle θ_*y*_ and wavelength λ_0_ as *k*_*y*_ =  sin θ_*y*_. The dispersion of the modes shows the linear TE and parabolic TM surface lattices resonance modes crossing at the Γ-point. The inset is a scanning electron micrograph of the gold nanoparticles on a glass substrate; the scale bar is 1 μm. (c) Total measured luminescence intensity of the sample as a function of pump fluence. (d, e) Unpolarized (d) and horizontally polarized (e) real-space images of the sample at 1.8 mJ cm^–2^ pump fluence.

When the nanoparticle arrays are combined with emitters (e.g., dye molecules), the SLR modes remain intact (weak coupling) for low emitter concentrations, while for high concentrations the strong-coupling regime is reached and the excitations transform into polaritons, that is, hybrids of the SLR modes and emitter excitations.^26^ The emitters may be pumped externally and serve as a gain medium. Three modalities of coherent emission have been observed so far: (1) lasing at the weak-coupling regime,^18,27^ (2) polariton lasing/condensation,^22^ and (3) Bose–Einstein condensation both at weak^24^ and strong couplings.^23^ The BEC requires thermalization consisting of multiple molecule–light absorption and emission processes associated with loss of energy to molecular vibrational degrees of freedom.^28^ The luminescence spectrum from the condensate shows a Bose–Einstein distribution, although the phenomenon is different from equilibrium BEC, as it occurs on the sub-picosecond scale. While polarization patterns have been observed previously for nanoparticle array lasers (e.g., see refs (21 and 29)), patterns associated with a nontrivial condensate phase have not been observed.

Here, we work with the strong-coupling BEC as introduced in ref (23). To reveal the fundamental properties of the condensate polarization structure, we use a highly symmetric geometry: the size and period of the lattice is the same in the *x*- and *y*-directions, and the particles are cylinders fully symmetric in the sample plane. We combine the array with a fluorescent solution of IR-792 at a concentration of 80 mM, which leads to strong coupling. The sample is pumped at 800 nm using left circularly polarized laser pulses generated with an ultrafast Ti:sapphire laser. The light radiated from the sample inherits the properties of the plasmonic excitations; therefore, the condensate can be characterized via real-space, spectral, *k*-space, and polarization-selective imaging. All of the real-space data shown here are luminescence collected after a single pump pulse; that is, they correspond to single shot realizations of the condensate (see the Supporting Information for details).

Figure 1c shows a distinct double-threshold behavior in the total measured luminescence as a function of the pump fluence. The first threshold corresponds to polariton lasing and the second one to strong-coupling BEC;^23^ we focus here on the latter regime. The real-space intensity profile of the condensate including all polarizations is non-uniform and *x*–*y* symmetric; see Figure 1d. With a linear polarizer in the *x*-direction, we obtain Figure 1e and a condensate peak at 1.406 eV, which differs from the Γ-point energy of the bare array (1.429 eV) due to strong coupling.^23^ When thermalizing toward the ground state (*k* = 0 band edge), the SLR excitations propagate due to their finite momentum **k**. This together with the finite size of the array leads to a non-uniform condensate profile, which in ref (23) was observed only in one direction (similar to Figure 1e) due to the use of a linearly polarized pump. The connection between the pump polarization and directionality is due to the stimulated nature of the thermalization process^23^ and the anisotropy of dipolar radiation of the nanoparticles (evident as the large TE–TM splitting for high *k*-values in Figure 1b). Namely, even when the pump is off-resonant from the SLR energy, it slightly excites the plasmonic dipole oscillations parallel to its polarization, and these in turn trigger the stimulated thermalization process to occur along the SLR dispersion branch with the same polarization and a specific direction. Here, the circularly polarized pump leads to propagation and non-uniform condensate density in both *x*- and *y*-directions.

Panels a–f of Figure 2 show the real-space intensity images of the pumped sample filtered using different polarizers. Panels a–b and e–f of Figure 2 reflect the underlying *x*–*y* symmetry of the system. Remarkably, the right and left circularly polarized images (Figure 2c,d) show complementary intensity patterns, with right circularly polarized light being emitted from the center and corners of the array, while luminescence close to the sides is mostly left circularly polarized. Panels g–l of Figure 2 are discussed later when we provide a model to explain the experimental findings. Note that right and left circular polarizations are superpositions of horizontal (here *x*) and vertical (*y*) linear polarizations 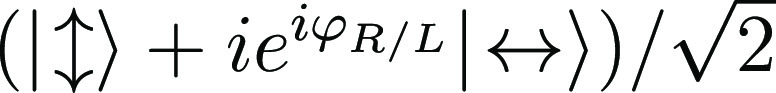, with the phase differing by π: φ_R_ = π, and φ_L_ = 0. This, together with the observed change from right to left circular polarization over the array, would hint toward having a *phase shift of the condensate* by π. Switching to a right circularly polarized pump causes the system to obtain an overall opposite phase, and the pattern formation in the circularly (Figure 2c,d) and diagonally (Figure 2e,f) polarized cases is changed such that c (e) and d (f) switch places. We have also used a diagonally polarized pump beam, which led to *different* patterns, due to the pump–polarization dependence of the stimulated thermalization; see Figure S2 and related discussion in the Supporting Information. This means that the patterns can be switched optically by femtosecond scale pulses. Note that the textures are not present in the polariton lasing regime; see Figure S3 in the Supporting Information.

**Figure 2:**
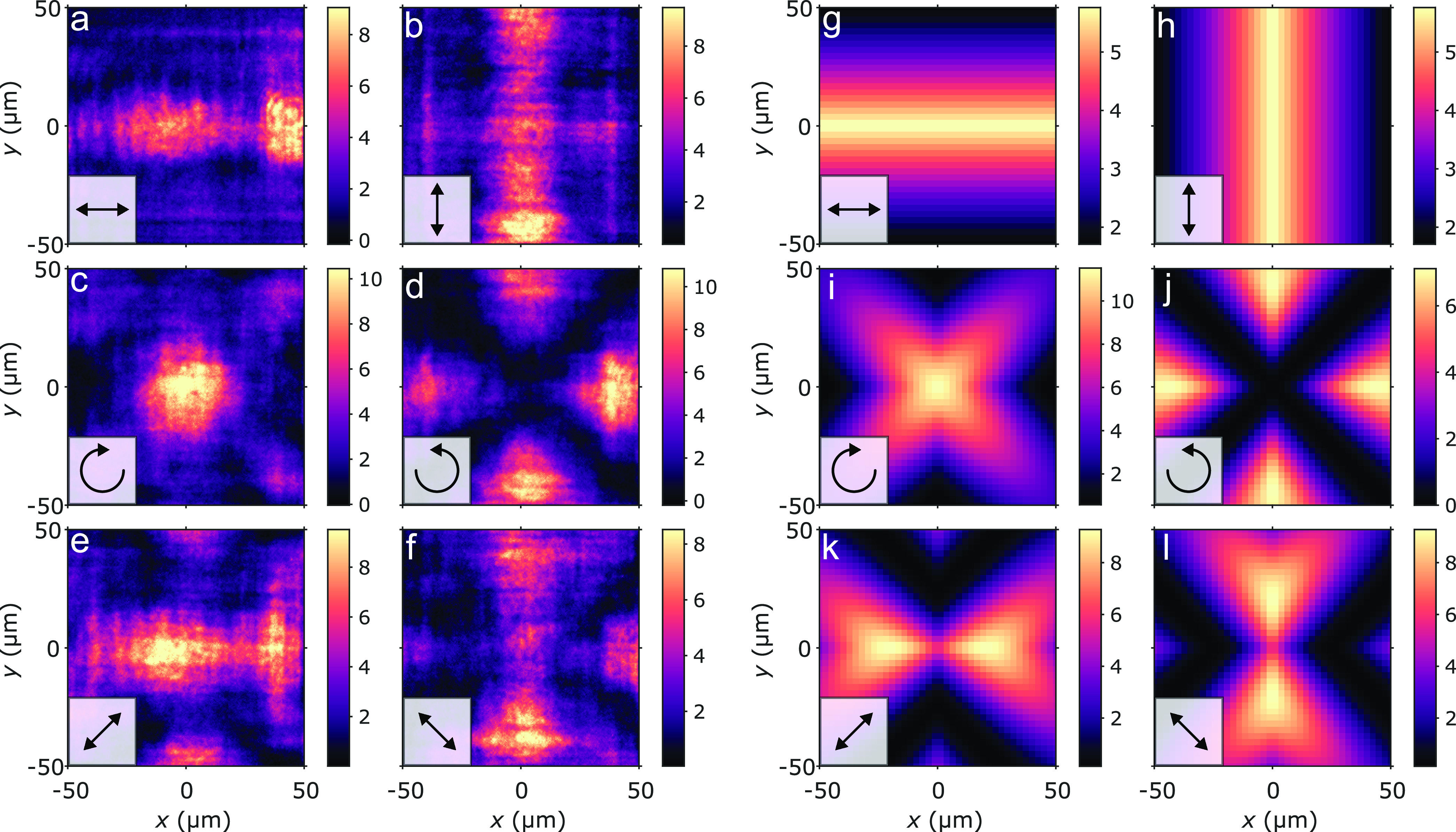
Real-space polarization patterns in a plasmonic condensate. (a–f) Sample emission intensities under left circularly polarized pumping with the fluorescence imaged through horizontal (a), vertical (b), right circular (c), left circular (d), diagonal (e), and antidiagonal (f) polarizers. These polarizers are illustrated with black arrows. (g–l) Electric field intensities obtained by a Jones vector model with polarizations corresponding to those in panels a–f.

In addition to the real-space data, we capture *k*-space (θ_*x*_, θ_*y*_) images of the luminescence (Figure 3a–f), which display striking similarities to their real-space counterparts: panels a–b and e–f of Figure 3 reflect the *x*–*y* symmetry, while the left and right circular polarization components (Figure 3c,d) have momentum distributions distinct from each other; i.e., they cannot be made the same by a rotation. Together, the real-space and Fourier domain data sets (Figure 2a–f and Figure 3a–f, respectively) allow us to reconstruct the phase profiles of the differently polarized emission patterns by utilizing the Gerchberg–Saxton phase retrieval algorithm (see the Supporting Information for details). Panels g–l of Figure 3 show the reconstructed real-space phase distributions. Remarkably, the images display *non-uniform phase profiles*: emission from the center of the plasmonic array has obtained an opposite phase compared to the emission from the edges. Particularly interesting is the left circularly polarized case which shows the opposite phase appearing between adjacent edges. Starting from right circular polarization 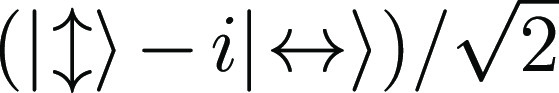 in the middle (Figure 3i), then moving toward the edges in the *x*-direction and adding a π phase shift to the |↔⟩ component as indicated by Figure 3g, one indeed obtains 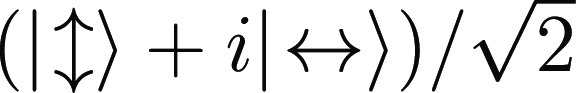, i.e., left circular polarization. In contrast, moving in *y* puts the minus sign in front of the 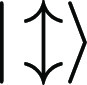 component, leading to left circular polarization *with an overall phase difference of π compared to the other edge*, namely, 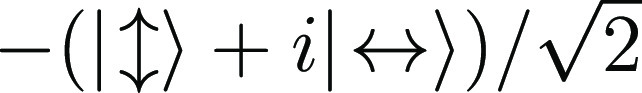, in full agreement with Figure 3j. This consistency between independently reconstructed images provides immediate proof of the robustness of the phase retrieval method. Note that, for a uniform condensate phase, one would expect an *intensity* pattern varying along *y* (*x*) as in Figure 2a(b) to lead to an extended feature along *y* (*x*) in the Fourier space, but Figure 3a(b) shows the opposite. This is, however, explained by the non-uniform *phase* varying along *x* (*y*) (Figure 3g(h)) since features in *k*-space are more strongly influenced by phase than amplitude.^30^

**Figure 3:**
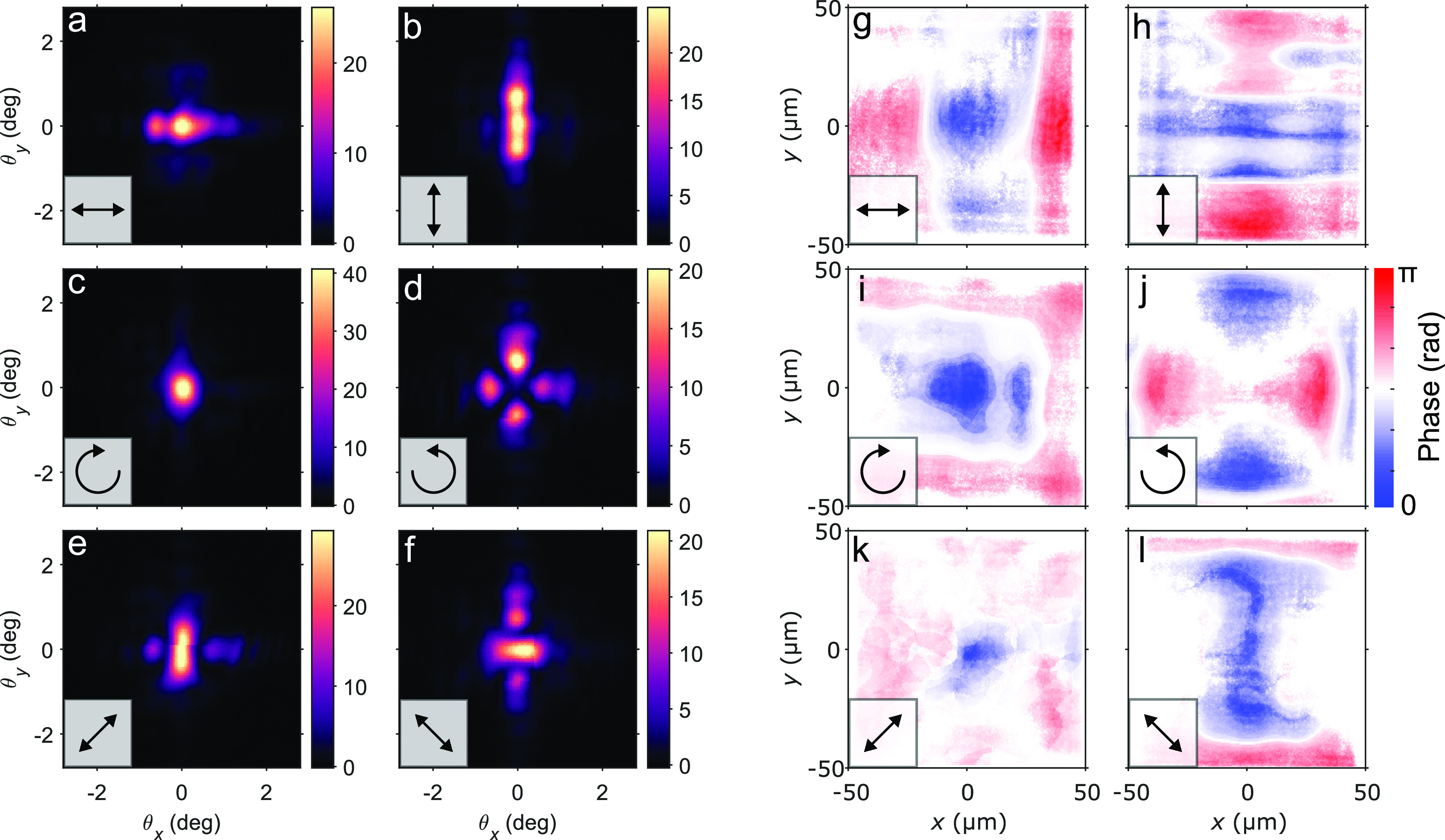
Reconstruction of phase in a plasmonic nanoparticle array. (a–f) Two-dimensional *k*-space intensity images of the spatially non-uniform emission patterns shown in Figure 2a–f. Here . (g–l) Real-space phase distributions reconstructed from the real and *k*-space data using the Gerchberg–Saxton algorithm. The images display the phase differences in the sample with an arbitrary overall phase.

On the basis of the non-uniform phase distributions obtained from the phase retrieval algorithm, we construct a simple yet highly effective model of the polarization states of the lattice plasmons. The array is modeled as a 2D grid of Jones vectors depicting the polarization of the emitted light. As a basis, we use the amplitudes *A*_H_ and *A*_V_, and phases φ_H_ and φ_V_ of the horizontally (*x*) and vertically (*y*) polarized electric field components. In the collective SLR lattice modes, the magnitude of a nanoparticle dipole oscillating in the *y*-direction (*x*-direction) decreases when moving from the center of the array toward the edges in the *x*-direction (*y*-direction). This is because the nanoparticles closer to the edges receive no radiation from outside the array. Figure 4a shows a schematic of our model. On the basis of the simple argument of nanoparticles receiving no radiation beyond the array boundaries, the decrease is linear and of a factor of 2. Alternatively, one can utilize the measured real-space intensity data in the model; this is discussed in the Supporting Information (see Figure S4). Motivated by the results of the phase reconstruction, we apply a linear approximation for the phase components: φ_H_ is varied from 0 to π and back as a function of *x* (purple line), and φ_V_ from π/2 to 3π/2 and back as a function of *y* (red line). The relative phase difference of π/2 between φ_H_ and φ_V_ is chosen to correspond to right circular polarization at the center of the array, as observed experimentally and anticipated from the pump polarization.

**Figure 4:**
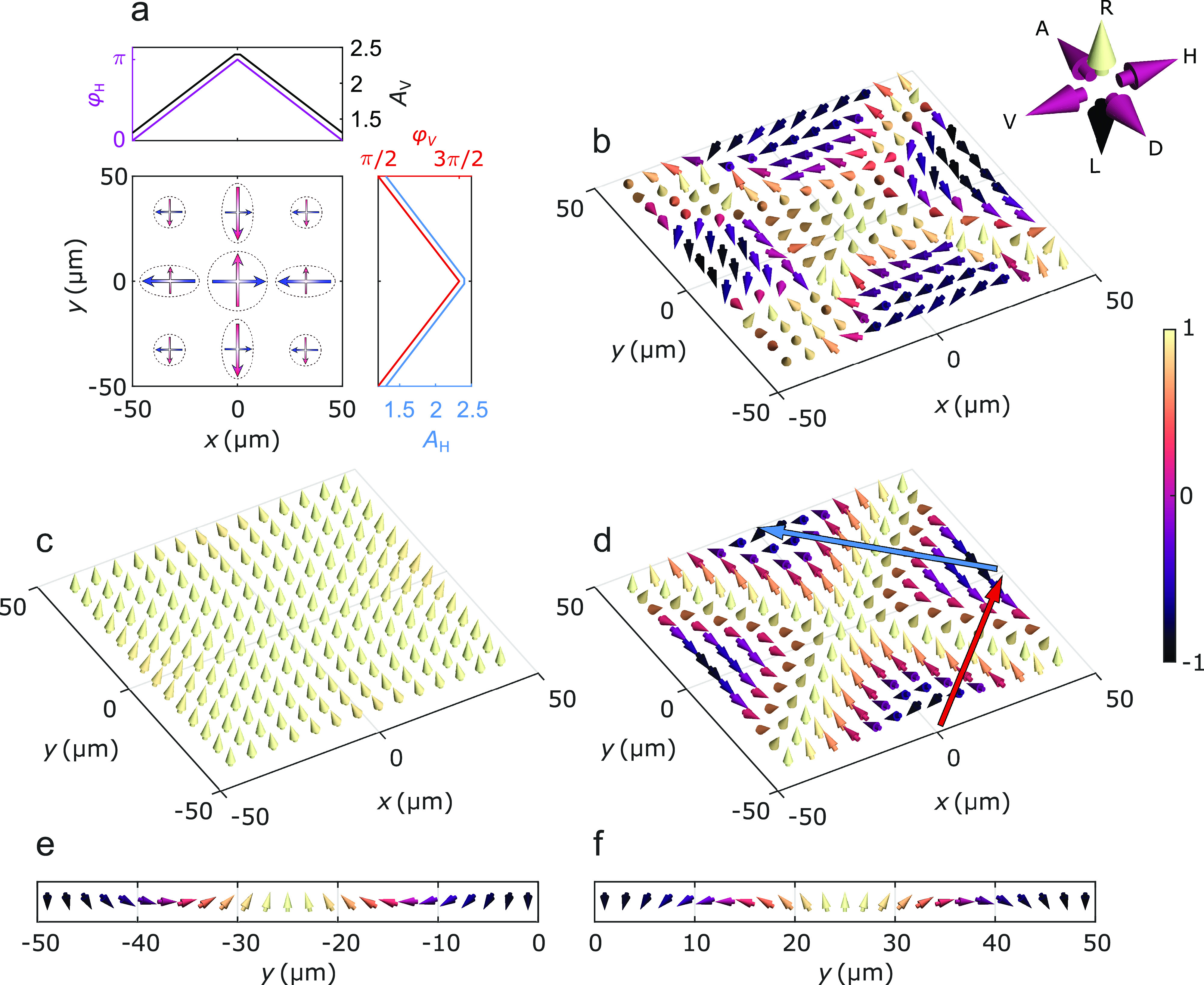
Jones vector grid and Stokes vector comparison. (a) Schematic of the array model of Jones vectors depicting the polarization states of the plasmonic condensate. The arrows in different points of the array depict the amplitudes (arrow length) and phases (arrow direction) of the horizontal (blue arrows) and vertical (red arrows) polarization components. The amplitude profile used in both components (black and blue lines) increases toward the sample center. The phase profiles are approximated linearly with a π-phase increase from the edges toward the array center (purple and red lines). (b–d) Stokes vectors illustrating the polarization state at different points on the array, calculated from the experimental real-space polarization patterns (b) and from theoretical dipole maps with constant (c) and non-uniform (d) phases. The relation between the polarization states and Stokes vectors is given in the arrow axes on the right. The length of each Stokes vector along the circular polarization axis is illustrated with a color scale, where −1 corresponds to left circular and +1 to right circular polarization. (e, f) Stokes vector winding along the red (e) and blue (f) arrows shown in panel d. The vectors are viewed parallel to the array surface toward negative *x*-values.

In order to compare the model to the measured real-space intensities, Jones calculus is applied to the 2D grid of Jones vectors, and the resulting electric field intensities of linear and circular polarization states are plotted in Figure 2g–l. Remarkably, the combination of non-uniform amplitude and phase distributions allows the model to qualitatively reproduce the real-space polarization patterns observed in the plasmonic condensate. Moreover, removing the π/2-phase difference between φ_H_ and φ_V_, which corresponds to diagonal polarization in the center of the array, indeed leading to images that are consistent with the experimental intensity patterns observed with a diagonally polarized pump beam (see Figure S2 in the Supporting Information). This demonstrates that the observed phase distributions can be switched by the polarization state of the pump.

So far, we have investigated projections of the condensate emission on different polarization components. Now we also calculate the Stokes vectors **S** = (*S*_1_, *S*_2_, *S*_3_), characterizing the pseudospin nature of polarization and how it evolves at different points on the array. The vector components are given by
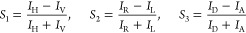
1where *I*_σ_ is the measured luminescence intensity and σ corresponds to the six different polarization states (horizontal (H), vertical (V), right circular (R), left circular (L), diagonal (D), and antidiagonal (A)). The pseudospin textures given by the experimental data, as well as those predicted by the model with uniform and non-uniform phase profiles, are shown in Figure 4b–d. The spin texture plotted with a uniform phase distribution (Figure 4c) does not produce the complex spin textures calculated from the experimental results (Figure 4b). In contrast, there is a striking resemblance between these experimentally observed pseudospin patterns and the model with the *non-uniform* phase distribution (Figure 4d), which demonstrates the importance of the discovered phase profiles in the system.

The observed pseudospin texture (Figure 4b) and the corresponding theory prediction (Figure 4d) show clear domain walls separating four regions with mostly left circularly polarized emission. Figure 4e shows the Stokes vector orientations following the red arrow across one of the domain walls in Figure 4d, and windings of 2π and ∼1.6π are observed along the R–D and R–H planes, respectively. We do not observe a full rotation along the R–H plane since the amplitudes of the horizontally and vertically polarized components are different at the edges. These windings are reversed in the adjacent domain wall (blue arrow) shown in Figure 4f, and, following a closed loop around the center of the sample, the total winding number becomes zero. Here, in a system with simple square lattice geometry, the pseudospin texture is of a nontopological nature. Given the broad tunability of the plasmonic nanoparticle array and the dependence of the phase profiles on the pump polarization, the creation of topologically nontrivial textures is a feasible goal.

In summary, we have observed polarization textures arising from an interplay between a structured optical medium and a non-uniform Bose–Einstein condensate phase. One ingredient of the textures is the finite size of the periodic array, which causes the nanoparticle dipoles to weaken toward the edges. This alone, however, would lead to nothing but unremarkable effects on the polarization properties (cf. Figure 4c). For the observed prominent domain wall structures (Figure 4e,f), an additional element is crucial: the *non-uniform phase* of the condensate. We revealed a zero-to-π phase change between the central and edge parts of the array with a Gerchberg–Saxton algorithm. In addition to being essential for explaining the textures, this constitutes the first experimental determination of a condensate phase by computational imaging, proposed earlier by theory.^31,32^ This achievement puts forward an attractive alternative to measurements of phase by interference, replacing complex experiments by a robust computational approach.

For our proof-of-concept demonstration of polarization textures, we used an *x*–*y* symmetric system. Future design possibilities include lattices with different geometry, size, and structure of the unit cell to realize new combinations of broken or competing symmetries, artificial gauge fields, and pseudospin–orbit coupling; different material choices such as dielectrics are also feasible.^33−35^ Importantly, the simple theoretical framework introduced here allows fast and intuitive planning of the desired textures. The expected qualitative behavior of the field intensities for different polarizations can be determined from the geometry of the lattice and straightforwardly generalized to higher order multipolar nanoparticle modes and more complex unit cells. Such an approach allows one to explore and plan polarization textures that various lattice configurations, together with different phase profiles, can produce. To exploit the condensate phase as a design degree of freedom, further studies are needed to understand its formation. Phase shifts of π are typically associated with wave function nodes, and the non-uniform intensity profile of our condensate might be associated with a soliton, for instance. However, for a reliable interpretation, existing theory describing the electronic, vibrational, and photonic degrees of freedom at the strong-coupling regime^36^ needs to be extended to include full thermalization dynamics and a finite sample size. Our results open new prospects for fundamental studies of vectorial (pseudospin) non-equilibrium condensates^25^ and topological photonics,^37^ as well as for tailoring bright coherent beams with complex polarization properties. The room temperature operation, straightforward sample fabrication, and ultrafast switching by the pump polarization are important assets. On-chip pumping would complete the list; combining plasmonic nanoparticle arrays with organic materials amenable to electrically induced gain^38^ is obviously a worthwhile future research direction.

## Supporting Information Available

Sample fabrication; experimental setup; phase retrieval; diagonal pumping; lasing regime images; simple theoretical model with experimental amplitude profiles (PDF)
