# Supplementary material for: Polarization and Phase Textures in Lattice Plasmon Condensates
Source: Nano Lett. 2021 Jun 2;21(12):5262–8. doi: 10.1021/acs.nanolett.1c01395 (PMC8289307; doi:10.1021/acs.nanolett.1c01395)
Supplement: Supplementary file 1 — nl1c01395_si_001.pdf [file nl1c01395_si_001.pdf]

Supporting Information: Polarization and phase textures in lattice plasmon condensates

Jani M. Taskinen, Pavel Kliuiev, Antti J. Moilanen, and Päivi Törmä*

Department of Applied Physics, Aalto University School of Science, P.O. Box 15100, Aalto, FI-00076, Finland

E-mail: paivi.torma@aalto.fi

Sample fabrication

Square arrays of Au nanoparticles are fabricated on borosilicate glass slides using electron beam lithography. A polymethyl methacrylate (PMMA) layer, which is spin-coated and baked solid on the glass substrates, is covered with 10 nm of evaporated aluminum and patterned using an electron beam. The aluminum layer is then removed using 50% AZ 351B developer and the PMMA layer is developed by immersion in 1:3 methyl isobutyl ketone:isopropanol solution. A thin (2 nm) titanium adhesion layer and a 50 nm gold layer are evaporated on the patterned slide, and excess PMMA and metal are removed by acetone lift-off. The array size is 100×100 μm^2, and the nominal periodicity 568 nm, which sets the Γ-point energy at ~ 1.44 eV. The height and diameter of the cylindrical nanoparticles forming the array are 50 nm and 105 nm, respectively.

The samples are prepared for measurements by sealing the nanoparticles between the substrate and a cover glass slide using a circular silicone isolator, whose thickness is 0.8 mm. For transmission measurements, the isolator is filled with index-matching oil; for condensation measurements,

it is filled with an 80 mM solution of IR-792 perchlorate dissolved in 1:2 dimethyl sulfoxide:benzyl alcohol mixture. The solvent has a matching refractive index with the glass slides ($n = 1.52$).

Experimental setup

A detailed schematic of the setup is shown in Fig. S1. The measurement setup can be used to measure both angle-resolved k-space spectra as well as real-space spectra with minor modifications. Light exiting the sample is collected using an infinity corrected objective (10x, 0.3 NA) together with a compatible tube lens. An optional polarizer may be placed after the tube lens to limit the measurement to a single polarization state. Here, the polarization is defined from the point of view of the source. A long pass filter (cutoff wavelength at 850 nm) is used in the detection path to filter out pump reflections. Light from the sample is spatially restricted to the nanoparticle array using an adjustable iris in front of the cameras.

In k-space measurements, the back focal plane of the objective is focused to the entrance slit of a spectrometer such that each point on the slit corresponds to a specific emission angle θ_y. The angle is related to momentum as $k_y = k_0 \sin\theta_y = \frac{2\pi}{\lambda_0}\sin\theta_y$, where k_0 and λ_0 are the free space wavenumber and wavelength, respectively. This allows the 2D charge-coupled device array inside the spectrometer to measure a spectrum at different values of k_y simultaneously. In addition, two fast complementary metal–oxide–semiconductor (CMOS) cameras are used to take direct real- and momentum-space images of the sample. The long pass filter limits the emission collected by the CMOS cameras to energies below 1.46 eV. In real-space measurements, an additional lens is placed after the tube lens, which causes the real-space image of the sample to be formed at the entrance slit of the spectrometer. In this case, each point on the slit corresponds to a specific y-coordinate of the sample.

In the condensation experiment, the sample is pumped optically using ultrafast laser pulses (50 fs pulse duration, 800 nm centre wavelength (1.55 eV energy)), that are left circularly polarized. However, as the pulses reflect off the sample, they are observed as right circularly polarized in the

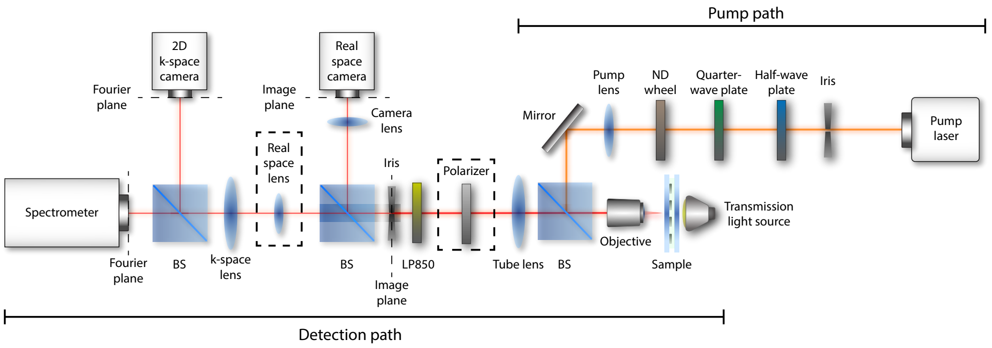

Figure S1: Schematic of the experimental setup used in the angle- and energy-resolved intensity measurements. The spectrometer and two cameras allow the setup to simultaneously measure the spectral information of the emitted light and capture Fourier and real-space images of the sample. Optional components are marked with dashed rectangles. Here, BS stands for beamsplitter and ND for neutral density, and LP850 refers to a longpass filter with a cutoff wavelength of 850 nm.

detection path. Since the repetition rate of our pump pulses is 1 kHz, setting the integration time of our CMOS cameras to 1 ms allows us to capture luminescence from a single realization of the condensate. An iris is used to spatially crop the pump beam, which is then focused on the nanoparticle array through the objective with the help of an additional pump lens. The polarization state of the pulses is controlled using motorized quarter- and half-wave plates on the pump path. Pump fluence is varied using a neutral density wheel. In transmission measurements, the array is illuminated using a broadband halogen light source.

Phase retrieval

Prior to phase retrieval, real- and k-space images shown in Figs. 2a-f and Figs. 3a-f were pre-processed using standard procedures.[1,2] Each dataset was centred in the computational domain. Fourier data were centred by finding a local maximum or a local minimum in the vicinity of the physical centre of the k-space intensity distribution. The centre of the real-space data was found by applying a watershed segmentation algorithm to the real-space intensity image, and computing the centre of mass of the segmented region. Real-space data were re-sampled to fulfill the relationship between the pixel sizes in the object and Fourier domains as set by the digital Fourier

transformation. The background noise (average 3100 counts in the object domain and 2200 counts in the k-space) was subtracted from each pixel; 3300 counts were subtracted from Fig. 2c and 2150 counts were subtracted from Figs. 3b-c, as this led to a better convergence.

The phase reconstruction was performed by the Gerchberg-Saxton phase retrieval algorithm.[3] The object-domain constraint was the square root of the processed real-space intensity distribution (Figs. 2a-f), and the Fourier constraint was the square root of the processed k-space intensity distribution (Figs. 3a-f). The linear oversampling ratio was ≈ 11 and thus fulfilled the oversampling condition.[4] In total, we performed 1000 independent reconstruction rounds with different initial random phase distributions in the k-space. The random phases were generated from a uniform distribution between -π and π. Each reconstruction round comprised 100 iterations, as this number of iterations was sufficient for the algorithm to converge. Only 5% of the reconstructed phase distributions having the lowest error metric in the k-space[5] were selected out of 1000 reconstructions and averaged following standard protocol.[6,7] For averaging purposes, the phase value in the centre of the computational domain was used as a reference, and accounted for an arbitrary global phase shift in the reconstructed images. The resulting reconstructed real-space phase distributions shown in Figs. 3g-l were weighted with the corresponding amplitude values (square root of the processed Figs. 2a-f) for illustration purposes.

Diagonal pumping

In addition to the circularly polarized pumping scheme described in the main text, we also investigate sample luminescence under diagonally polarized pumping. The resulting real-space images are presented in Fig. S2, where the black arrows illustrate the polarization state of luminescence. The overall measured intensity is lower compared to circularly polarized pumping, while the vertically and horizontally polarized components do not show a clear accumulation of SLR excitations to the centre of the sample. A comparison between Figs. 2 and S2 shows that, although both pump configurations show the same assortment of patterns, the textures associated with circular and di-

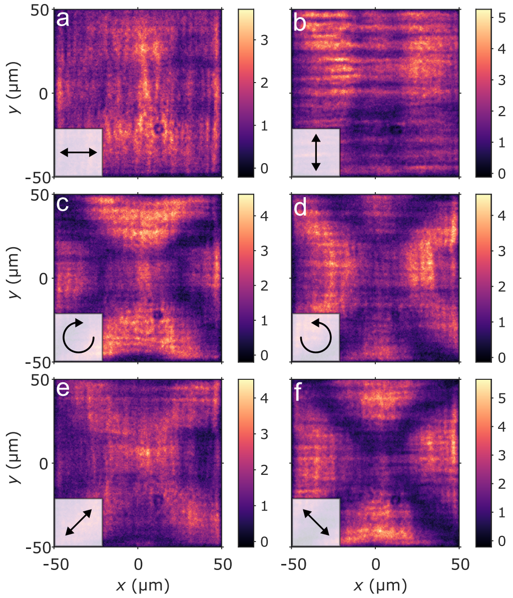

Figure S2: Polarization patterns under diagonal pumping. (a-f) Real-space intensities emitted by the diagonally pumped sample measured through horizontal (a), vertical (b), right circular (c), left circular (d), diagonal (e) and antidiagonal (f) polarizers. Similarly to the case of circularly polarized pumping, the peak of horizontally polarized emission is found at 1.406 eV.

agonal polarization states are switched when we change the pump polarization: if patterns d and c are swapped with e and f in Fig. 2, we reach the arrangement of textures in Fig. S2. The phase delay between the vertical and horizontal components of the pump is inherited to the plasmonic excitations and affects the observed polarization textures. A plausible explanation is the following. The pump beam is not resonant with the plasmonic modes and thus mainly excites the molecules, however, it also drives off-resonant weak excitations in the nanoparticles. These excitations reflect the pump polarization: linear polarization causes dipolar oscillations in the direction of the polarization, whereas circular and diagonal polarizations induce oscillations in the x- and y-directions with a specific phase delay. These plasmonic excitations then trigger the stimulated thermalization process, and thereby information about the pump polarization is inherited to the condensate. Initiation of the condensation process by stimulated rather than spontaneous emission is in accordance with the ultrafast timescales and stimulated nature of the thermalization that were observed in Ref. 6.

Lasing regime images

Figure S3 shows the polarization filtered real-space images of the sample emission at the onset of the lasing regime. Polarization textures are not present as the circularly (c,d), diagonally (e) and antidiagonally (f) polarized images are nearly identical and their features follow a simple combination of the horizontally (a) and vertically (b) polarized images.

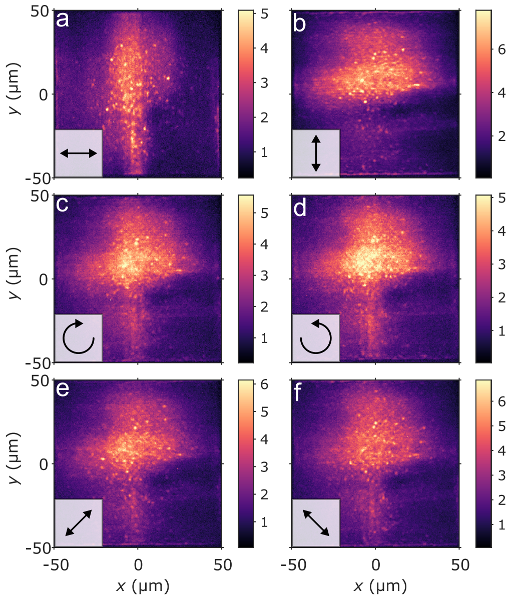

Figure S3: Sample emission at the onset of lasing. (a-f) Real-space intensities emitted by the sample measured through horizontal (a), vertical (b), right circular (c), left circular (d), diagonal (e) and antidiagonal (f) polarizers. The pump beam is left circularly polarized and the pump fluence is 0.4 mJcm^{-2}. Here the integration time of the CMOS camera was increased to 300 ms due to the low emission output of the lasing action.

Simple theoretical model with experimental amplitude profiles

The condensate intensity profiles are non-uniform as shown in Fig. 1d without polarizers, and in Fig. 1e and Figs. 2a-f with polarization resolved. To illustrate this more clearly, we take the intensity values between $x = 0...50$ μm in Fig. 2b and average them over the array in the y-direction: such profiles are shown in Fig. S4a. The origin of the non-uniform condensate intensity is in the finite size of the lattice, but the profiles are not linear. This is plausible, as the condensation process

involves non-linearities (stimulated processes and saturation-induced effective photon-photon interactions[8]). One may utilize the experimentally obtained intensity profiles in the theoretical model instead of the linear approximation for the decrease of electric field components; Figs. S4b-g show that it leads to an even closer match between the experiment and the model. However, in the main text we chose to utilize the linear approximation in order to highlight the predictive power of the simple model.

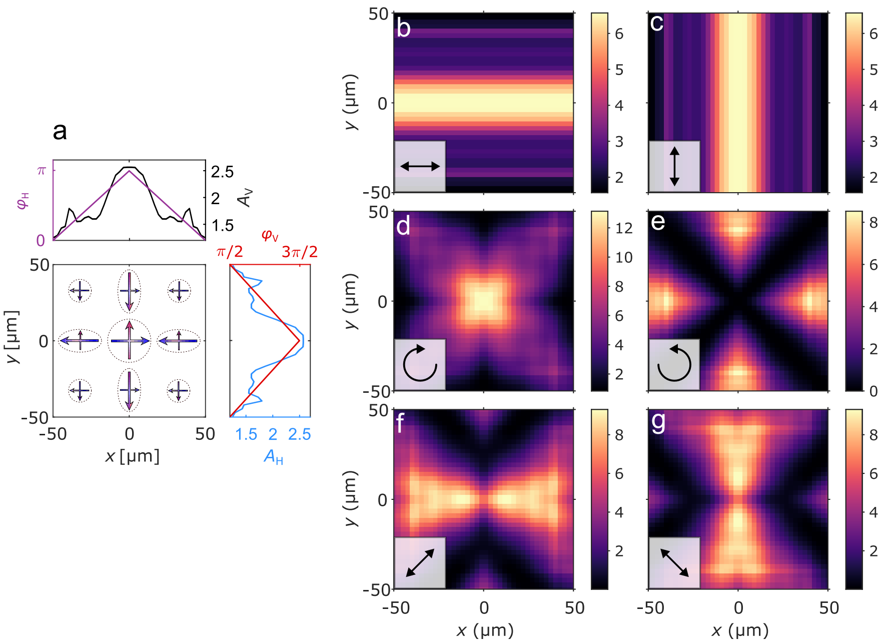

Figure S4: Jones vector model with fitted amplitude profiles. (a) Modified version of the theoretical model shown in Fig. 4a. The linearly approximated amplitude profiles are replaced with averaged amplitude values from Fig. 2b. (b-g) Electric field intensities obtained by the Jones vector model presented in (a) with horizontal (b), vertical (c), right circular (d), left circular (e), diagonal (f) and antidiagonal (g) polarizers.

References

(1) Kliuiev, P.; Latychevskaia, T.; Osterwalder, J.; Hengsberger, M.; Castiglioni, L. Application of iterative phase-retrieval algorithms to ARPES orbital tomography. *New J. Phys.* **2016**, *18*, 093041.

(2) Latychevskaia, T. Iterative phase retrieval in coherent diffractive imaging: practical issues. *Appl. Opt.* **2018**, *57*, 7187–7197.

(3) Gerchberg, R. W.; Saxton, W. O. A Practical Algorithm for the Determination of Phase from Image and Diffraction Plane Pictures. *Optik* **1972**, *35*, 237–246.

(4) Miao, J.; Sayre, D.; Chapman, H. N. Phase retrieval from the magnitude of the Fourier transforms of nonperiodic objects. *J. Opt. Soc. Am. A* **1998**, *15*, 1662–1669.

(5) Fienup, J. R. Reconstruction of an object from the modulus of its Fourier transform. *Opt. Lett.* **1978**, *3*, 27–29.

(6) Latychevskaia, T.; Chushkin, Y.; Zontone, F.; Fink, H.-W. Imaging outside the box: Resolution enhancement in X-ray coherent diffraction imaging by extrapolation of diffraction patterns. *Appl. Phys. Lett.* **2015**, *107*, 183102.

(7) Kliuiev, P.; Latychevskaia, T.; Zamborlini, G.; Jugovac, M.; Metzger, C.; Grimm, M.; Schöll, A.; Osterwalder, J.; Hengsberger, M.; Castiglioni, L. Algorithms and image formation in orbital tomography. *Phys. Rev. B* **2018**, *98*, 085426.

(8) Väkeväinen, A. I.; Moilanen, A. J.; Nečada, M.; Hakala, T. K.; Daskalakis, K. S.; Törmä, P. Sub-picosecond thermalization dynamics in condensation of strongly coupled lattice plasmons. *Nat. Commun.* **2020**, *11*, 3139.
